# Jammer Classification in GNSS Bands Via Machine Learning Algorithms

**DOI:** 10.3390/s19224841

**Published:** 2019-11-06

**Authors:** Ruben Morales Ferre, Alberto de la Fuente, Elena Simona Lohan

**Affiliations:** 1 ITC Faculty, Department of Electrical Engineering, Tampere University, 33720 Tampere, Finland; ruben.moralesferre@tuni.fi; 2 GMV Aerospace & Defence, GNSS Department, 28760 Madrid, Spain; afuente@gmv.com

**Keywords:** jamming, Global Navigation Satellite Systems (GNSS), classification, image processing, deep learning, Convolutional Neural Networks (CNN), Support Vector Machines (SVN)

## Abstract

This paper proposes to treat the jammer classification problem in the Global Navigation Satellite System bands as a black-and-white image classification problem, based on a time-frequency analysis and image mapping of a jammed signal. The paper also proposes to apply machine learning approaches in order to sort the received signal into six classes, namely five classes when the jammer is present with different jammer types and one class where the jammer is absent. The algorithms based on support vector machines show up to 94.90% accuracy in classification, and the algorithms based on convolutional neural networks show up to 91.36% accuracy in classification. The training and test databases generated for these tests are also provided in open access.

## 1. Introduction and Motivation

Jamming threats are increasing in the Global Navigation Satellite System (GNSS) bands. According to recent Eurocontrol Voluntary ATMincident reports [1], the number of Global Positioning System (GPS) outages has been increasing exponentially over the past five years, and these numbers are predicted to increase further. The main cause of GPS outages is the presence of jamming signals in the GPS frequency bands, i.e., L bands around 1.5 GHz carrier frequency. A jamming signal can be typically divided into human-made or channel based. Human-made interferences can be split into intentional (e.g., jamming or spoofing) or unintentional (e.g., interference produced by other systems such as inter-modulation products or radio resource allocation). Channel based interferences involve phenomena such as multi-paths, atmospheric scintillation, or fading. Jamming then can be defined as an intentional (usually narrowband) interference in the wireless bands of interest, with received powers of several orders of magnitude higher than the useful received powers, in this case the powers of the GNSS signals. The high received power differences between the jammers and the GNSS signals are due to the fact that the jammers are typically placed on Earth or in the vicinity of the Earth’s surface (e.g., drone installed jammers), while the GNSS satellites are about 19–23 thousands km above the Earth’s surface; thus, the path-loss attenuation is significantly higher for the GNSS signals than for the jammer signals.

Given the increased prevalence of interferences in GNSS bands, the focus in the GNSS literature in recent years has shifted towards integrity methods and procedures to deal with jamming and other interference signals, such as meaconing and spoofing. There are several jamming management solutions, such as detection, mitigation, localization, or classification. The focus in the literature so far has encompassed all-but-jamming classification solutions. For example, jamming detection solutions were reported in [2,3,4]; jamming mitigation algorithms can be found in [2,5,6,7]; and jamming localization was addressed in [3,8]. However, very little effort has been dedicated so far to the jamming classification algorithms, which are also an important stage towards an efficient interference management solution. According to [9], there are several main classes of jammers. The classification we adopt here encompasses five main jammer classes, namely: (i) Amplitude Modulated (AM) jammers, (ii) chirp jammers, (iii) Frequency Modulated (FM) jammers, (iv) pulse jammers or Distance Measurement Equipment (DME)-like jammers, and (v) Narrow Band (NB) jammers. There is also a sixth category of Wide Band (WB) jammers, but this is typically very hard to detect or classify as the signal characteristics in the time and frequency domain are very similar in the presence and in the absence of jammers. This similarities are due to the fact that the GNSS signal is also a wideband signal, having a noise-like power spectral density. In our paper, we will focus on the classification of the five categories mentioned above, (i) to (v), in addition to being able to classify correctly the absence of jammers (i.e., no jammer present). To the best of the authors’ knowledge, currently, there is no published work on the classification results of jamming types in GNSS bands. Related works deal with NB jammer classification [10,11] and chirp signal classification [12], but only in the context of radar systems.

The paper’s contributions are as follows: (i) proposing to treat the jammer classification as a black-and-white image classification problem, based on the spectrograms computed at the GNSS receiver from the Intermediate Frequency (IF) samples; (ii) proposing efficient Support Vector Machines (SVM) and Convolutional Neural Network (CNN) based classifiers with classification accuracy of 90% or more; (iii) offering to the research community an open-access comprehensive database of clean and jammed GNSS signal spectrograms for a variety of Carrier-to-Noise Ratio ($C/N_{0}$) and Jammer-to-Signal Ratio (JSR) conditions.

The rest of the paper is organized as follows: Section 2 gives a mathematical modeling of the considered jammer types; Section 3 formulates the jammer classification into an image classification problem by using a short-time short-frequency decomposition of the incoming signal; Section 4 gives an overview of the machine learning algorithms we propose to use for the classification purposes; Section 5 presents the results based on SVM and CNN; Section 6 gives the references to the repository where we made our data public; and Section 7 summarizes the findings.

## 2. Jamming Types

If we consider r(t) as the signal reaching a GNSS receiver, r(t) can be modeled generically as:

(1) r(t)=g(t)+j(t)+s(t)+w(t),

where g(t) represents the signals of interest, coming from the GNSS constellation of satellites, j(t) is a possible jamming signal including adjacent-band interference (e.g., harmonics from other systems close to GNSS bands), and s(t) denotes a possible spoofing signal. The background noise over the wireless propagation channel is modeled as AWGN and denoted by w(t). In the absence of jamming, j(t)=0, and in the absence of spoofing, s(t)=0. In our studies, we will concentrate on the case of no spoofers, but jamming is present, i.e., j(t)≠0 and s(t)=0.

As can be found in the literature [9,13], the jamming signals j(t) can be typically divided into several jamming classes, typically according to the difficulty in detecting them. Below, we present the mathematical models of the jammers considered in our study.

### 2.1. AM Jammers

This class of jammers contains the simplest and at the same time the most studied jammer types, and they are also referred to as Continuous Wave (CW) jammers. We would like to emphasize that the AM noise jammers are different from the AM/CW simple jammers described here; our AM jammers refer to single- or multi-tone sinusoidal signals, weighted with various amplitudes, as shown below in Equation (2). AM jammers can be single-tone or multi-tone. An AM-modulated jammer can be modeled using Equation (2) and consists of k=1,…,K single tones (K=1 for the single-tone case, and K>1 for the multi-tone case). It is characterized mainly by three parameters, namely $P_{J_{k}}$, $f_{J_{k}}$, and $\theta_{J_{k}}$, which stand for the power at the antenna and the corresponding carrier frequency and phase of the $k^{\mathrm{th}}$ jammer component, respectively.

(2) $$j\left(t\right)=\sum_{k=1}^{K}\sqrt{P_{J_{k}}}\exp\left(j\left(2\pi f_{J_{k}}t+\theta_{J_{k}}\right)\right)$$

### 2.2. Chirp Jammers

This category contains jammer signals whose frequency is modulated linearly over time. They are generated by sweeping linearly their frequency over a certain frequency range and a certain time period, after which the process is started again at the initial frequency. These saw-tooth chirp jammers can be modeled by Equation (3). The parameters shown in Equation (3) are: the jamming power $P_{J}$, the starting frequency of the sweep $f_{J}$ (at time $T_{\mathrm{swp}}=0$), the minimum and maximum frequency sweep $f_{\min}$ and $f_{\max}$, and the sweep period $T_{\mathrm{swp}}$, which is the time it takes the jammer to sweep from $f_{\min}$ to $f_{\max}$. The variable b=±1 is a flag determining if we have an up-chirp (b=1) or a down-chirp (b=−1); $\theta_{J}$ denotes the initial phase of the jammer; and $f_{q}\left(t\right)=2\pi f_{J}t+\pi b\frac{\left(f_{\max}-f_{\min}\right)}{T_{\mathrm{swp}}}t^{2}$ is the instantaneous frequency of the jamming signal.

(3) $$\begin{array}{c} \begin{array}{ccc} j(t) & = & \sqrt{P_{J}}\cdot \exp\left(j\left(2\pi f_{J}t+\pi b\frac{\left(f_{\max}-f_{\min}\right)}{T_{\mathrm{swp}}}t^{2}+\theta_{J}\right)\right)\\ & = & \sqrt{P_{J}}\cdot \exp\left(j\left(f_{q}\left(t\right)t+\theta_{J}\right)\right) \end{array} \end{array}$$

### 2.3. FMJ ammers

FM jammers can also be single-tone or multi-tone. An FM jammer can be modeled using Equation (4), for which the carrier frequency is time dependent. Equation (4) incorporates a parameter $\beta_{k}$ into the signal model, which is the modulation index of the $k^{\mathrm{th}}$ tone. For the single-tone case, K=1.

(4) $$j\left(t\right)=\sum_{k=1}^{K}\sqrt{P_{J_{k}}}\exp\left(j\left(2\pi f_{J_{k}}t+\beta_{k}\cdot \sin\left(2\pi f_{J_{k}}t\right)\right)\right)$$

### 2.4. Pulse Jammers or DME-Like Jammers

These are jamming signals that are active only during repetitive periods of time with an active period of a pulse called the “duty cycle”. Pulse jammers can be modeled using Equation (5), where $p_{\tau }\left(t\right)$ is a rectangular pulse of width τ (the duty cycle), $f_{r}$ is the pulse repetition frequency, δ(t) is the Dirac pulse, and ⊗ is the convolution operator.

(5) $$j\left(t\right)=\sqrt{P_{J}}p_{\tau }\left(t\right)⊗\sum_{k=1}^{K}\delta \left(t-\frac{k}{f_{r_{k}}}\right)\cdot \exp\left(j2\pi f_{J_{k}}t\right)$$

### 2.5. NB Jammers

The narrowband noise jammers are jammers that span a narrow band of the signal spectrum, e.g., by sending a higher power signal in that spectral region. Such jammers can be modeled using Equation (6), where β is the modulation index and n(ζ) represents a stationary random process with zero mean and $\sigma_{\zeta }^{2}$ variance.

(6) $$j\left(t\right)=\sqrt{P_{J}}\cos\left(2\pi f_{J}t+\beta \int_{0}^{t}n\left(\zeta \right)d\zeta +\theta_{J}\right)$$

## 3. Novel Problem Formulation of Jammer Classification

**Figure 1:**
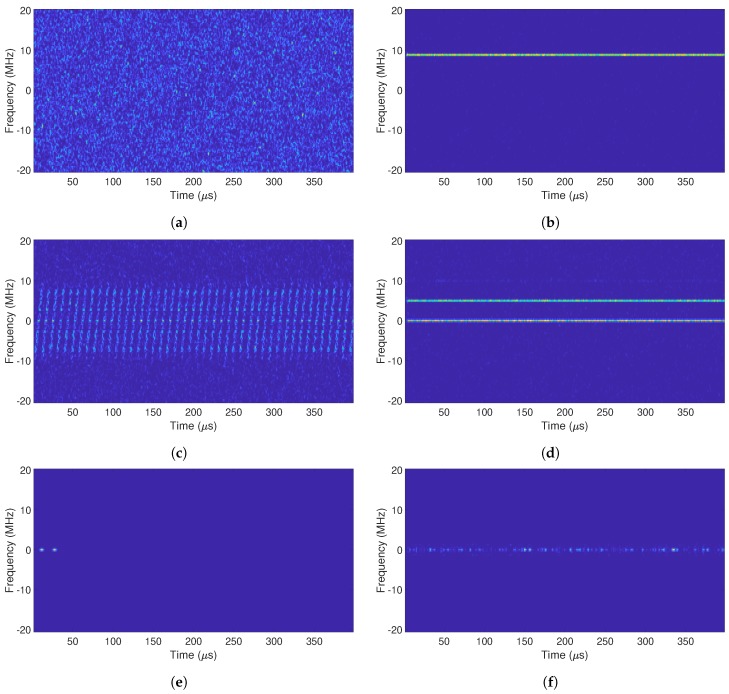
Spectrograms of common jamming signals in the baseband. All graphs contain a signal mixture of the jamming signal and the GPS L1 signal, where the *C/N_0_* is 45 dB and the JSR is 50 dB. (**a**) No jammer. (**b**) AM jammer. (**c**) Chirp jammer. (**d**) FM jammer. (**e**) Pulse jammer. (**f**) NB jammer.

The different jammer signals described in Section 2 affect in different manners the received signal r(t). The time-frequency information of r(t) carries significant information about the possible presence of jammers. As some of the jammer types are non-stationary, as is the case of the chirp jammers, the best time-frequency modeling is a short-time short-frequency transform such as the signal spectrogram. In order to compute the spectrogram, first we generate a short signal of 1 ms in duration, containing jamming and the useful signal or the useful signal alone. The short-term short-frequency spectrogram transform relies on parameters chosen as a trade-off between achieving a good resolution for all the considered jammer types and maintaining a low complexity. Therefore, the spectrogram has a window length of 128 samples with an overlap of 120 samples and an Fast Fourier Transform (FFT) size of 128 samples. Figure 1 gives examples of different spectrograms that can be obtained for the considered jamming signal types. The full-colored spectrograms are depicted here, while their black-and-white exemplification will be shown in Section 5.

To obtain the plots in Figure 1, the $C/N_{0}$ and JSR were set to 45 dBHzand 50 dB, respectively, in order to have a clear view of the jammer presence. For all the simulations carried out, the chosen GNSS constellation and frequency band was GPS L1, which corresponds to 1.57542 GHz. In Figure 1a, the spectrogram of the clean GNSS is given (i.e., no jammer present), while the sub-plots, Figure 1b–f, show examples of the spectrograms for the five considered jammer signals. In the absence of jamming, we observed a WB signal composed basically of noise, since there was no other contribution than the GNSS signal itself, which is a low-power Code Division Multiple Access (CDMA) signal and thus noise-like. On the contrary, for the rest of the scenarios in Figure 1b–f, a certain contribution from a certain jammer type can be noticed. For example, in Figure 1b or Figure 1d, we notice horizontal straight lines at a certain frequencies, which correspond to the frequencies of the AM or FM tones’ contribution, respectively, of the jamming signal j(t). In Figure 1c, we observe the typical spectrogram of a saw-tooth signal showing the sweep range and sweep periods of an up-chirp signal, as a typical saw-tooth signal. Figure 1e shows a double-pulse signal active before 50 μs. Finally, in Figure 1f, the spectrogram shows that there exists a certain contribution of band-limited noise, which only affects a certain (narrow) bandwidth of the signal r(t).

**Figure 2:**
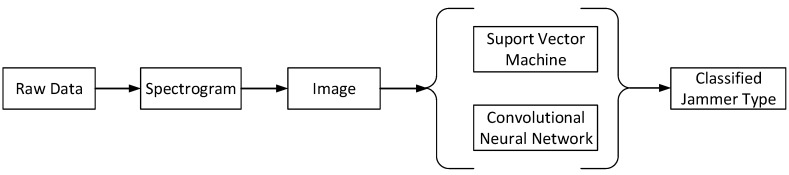
Block diagram of the proposed methodology.

In this article, we propose a methodology in order to identify and classify the different jammer types based on the analysis of such a spectrogram image. The methodology is depicted in the block diagram of Figure 2. The raw data corresponding to r(t) and containing both the GNSS and interference signals (if any) were used first to produce a spectrogram, as the ones shown in Figure 1. The spectrogram image was then saved as a black-and-white image (monochrome .bmp image) in order to reduce the number of (irrelevant) features. It was saved with a resolution of 515 × 512 pixels and 600 DPI. After that, one of the chosen machine learning algorithms was applied. The choice of a black-and-white image instead of the colored one was motivated by our empirical studies, as well as by the intuition that SVM classifiers are known to work better with binary images than with multi-level images.

The choice of the machine learning algorithms for classification was also based on literature studies. We selected SVM and CNN, as described in Section 4, based on their reportedly good performance in image classification problems, as described in Section 4. These algorithms classify the images according to the different features they can extract from them into six different classes, namely one of the five jammer class types described above or no-jammer class.

## 4. Machine Learning Algorithms

In this section, the implemented machine learning methods to perform the classification are briefly described. For the SVM method, proposed for example in Vapnik [14,15], we basically extracted the image features from the black-and-white images by using the method called Bag of Features (BoF) [16]. These features were used as input data to the SVM classifier. The SVM classifier included two stages: a training stage based on training data available and a classification stage based on new data (called test data). The second studied classifier was the CNN classifier, based on multi-layer neural networks [17]. The reason to choose SVM and CNN as classifiers was their excellent performance for various classification problems reported in the recent literature [18,19,20].

### 4.1. Image Features: Bag of Features (BoF)

A bag of features method consists of representing images based on extracting local features. The name comes from the Bag of Words (BoW) representation used in textual information retrieval [16]. Analogous to BoW, by which we can represent a certain document as a normalized histogram of word counts and build-up a word dictionary, by using BoF, we can do a similar thing, but using image features instead of words. The representative features of the images were gathered into clusters, which contained similar features. Every time a new representative feature was detected, it was matched to the nearest cluster feature from the visual vocabulary. At a high level, the procedure for generating a bag of features image representation can be summarized into three steps [16]:

1. Vocabulary building: First of all, the features from all the training images were extracted. These features were stored in a “visual vocabulary” dictionary, where each feature represented a “visual word” or “term”.
2. Term assignment: After extracting the features, they were gathered into clusters. The clusters collected the closest terms in the vocabulary dictionary in order to reduce the complexity.
3. Term-vector generation: The term vector was generated by recording the counts of each term that appeared in the image to create a normalized histogram counting the times it was repeated in the cluster. This term vector was the bag of features representation of the image.

The set of features contained in the term vector were used by SVM to classify the data into the different classes.

### 4.2. Support Vector Machines (SVM)

**Figure 3:**
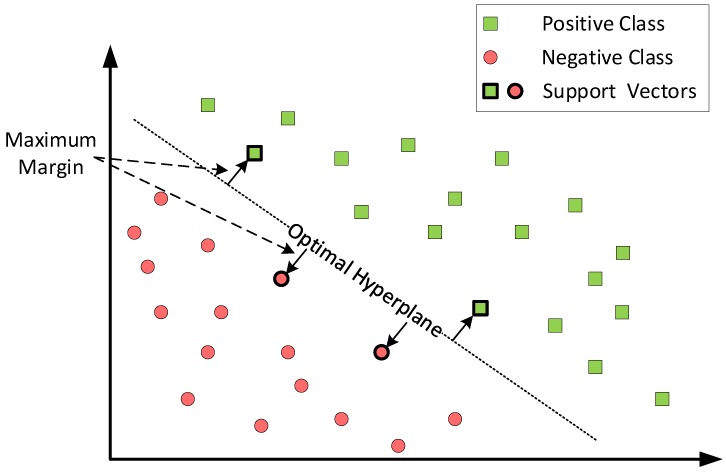
SVM binary classification.

SVM consists of a set of supervised learning methods that can be used for classification, regression, and/or ranking. Supervised learning means that the user must take part during the training process of SVM. The user must label the training data previous step of training SVM. SVM uses machine learning theory as a principle of operation to learn and to perform classification. SVM performs classification by transforming (if needed) the original training data into multidimensional space and constructing a hyper-plane with it. The established hyper-planes will split the different classes to classify. The hyper-plane with the maximum distance between support vectors, which are the closest data points to the hyper-plane, is the hyper-plane used to classify, and it is called the optimal hyper-plane. Therefore, our goal was to maximize the margin between the edge data points and the hyper-plane in order to be able to classify with the minimum miss-classification error. Figure 3 shows a two-class separation scenario. The green squares represent the positive class, and the red dots represent the negative class. The bold green squares and red dots are the support vectors, which were the samples that were the most difficult to classify because they were at the border between both data types and were used to determine which was the optimal hyper-plane. The optimal hyper-plane in Figure 3 is drawn with a dotted line, and it was the hyper-plane with the maximum distance between the different support vectors. In this example, the classification can be easily performed since the data were linearly separable, and we only had two classes. For more than two classes, a graphical representation is harder to draw, but the reasoning and extension to N classes is straightforward (N=6 in our case).

In order find the optimal hyper-plane, we could start defining a hyper-plane as:

(7) $$w^{T}x+b=0$$

where $w^{T}$ is the normal vector to the hyper-plane containing the weights toward the different data points, x contains the points defining the hyper-plane, and b is a bias constant. Our dataset was composed by $z=z_{1},z_{2},\ldots ,z_{m}$ and $y_{i}\in -1,1$ being the class label of z, which was −1 or one for the negative and positive class of the example, as shown in Figure 3, respectively. We can now define the decision boundary, which should classify all points correctly as:

$$\begin{array}{c} w^{T}z+b\geq 1\;if\;y_{i}=1\\ w^{T}z+b\leq -1\;if\;y_{i}=-1 \end{array}$$

Among all the hyper-planes separating the data z, there exists an optimal hyper-plane yielding the maximum margin of separation between the classes to classify. This optimal hyper-plane can be found by optimizing Equation (7) as:

(8) $$\max_{\left\{w,b\right\}}\;\min\{\|x-z\|:x,z\in {ℜ}^{N},\left(w^{T}x+b=0\right)\}$$

The weight vector w will consider the values of z closer to the hyper-plane with a higher value, the closest ones of each class being the support vectors, as is shown in Figure 3.

To find the optimal hyper-plane, different methods can be used. Some of the most popular are the Iterative Single Data Algorithm (ISDA) [21], L1 soft-margin minimization by Quadratic Programming (L1QP) [22], and Sequential Minimal Optimization (SMO) [23].

**Figure 4:**
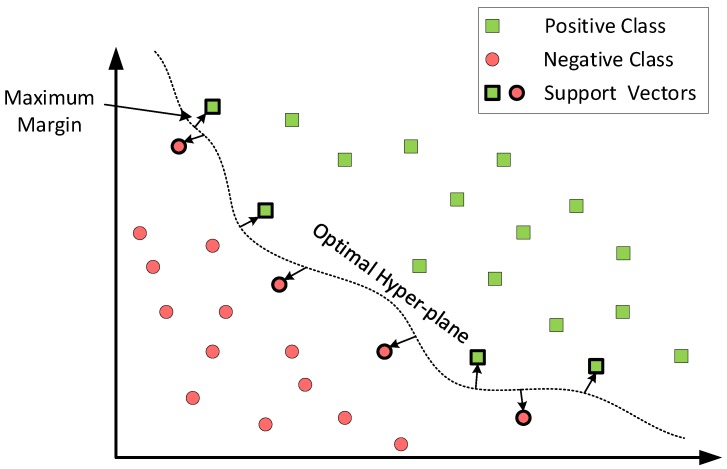
SVM binary classification with non-linear separation.

Typically, most classification tasks cannot be separated as easily as in Figure 3, and often, more complex or higher order structures are needed in order to make an optimal separation, such as is shown in Figure 4. When we could not find a linear separator, the data points were typically projected into a higher dimensional space where the data points became linearly separable. This transformation was carried out with functions called kernels. Basically, a kernel maps the non-linear separable dataset into a higher dimensional space where we can find a hyper-plane that can separate the samples linearly. One example of this could be to map the 2D data representation into a 3D representation. It might happen that in 3D space, the data can be easily separable by a simple straight line, as in Figure 3.

There are multiple kernel types that we could use to transform the data. Some of the most popular ones are the linear kernel, the polynomial kernel, and the Radial Basis Function (RBF) kernel.

The linear kernel is defined as:

(9) $$K\left(z_{i},z_{j}\right)=z_{i}\cdot z_{j}$$

where $z_{i}$ and $z_{j}$ are two different support vectors and K(·) is the kernel function.

The polynomial kernel could be expressed as:

(10) $$K\left(z_{i},z_{j}\right)={\left(z_{i}\cdot z_{j}+c\right)}^{d}$$

Finally, the RBF kernel is defined as:

(11) $$K\left(z_{i},z_{j}\right)=\exp(-\gamma \|z_{i}-z_{j}{\|}^{2})$$

The kernel choice in our studies is discussed in Section 5.2.

### 4.3. Convolutional Neural Network (CNN)

**Figure 5:**
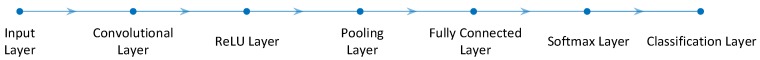
Layer architecture list.

A CNN or Convolutional Neural Network (ConvNet) consists of a sequence of layers, where every layer transforms the data into a simplified version through a differentiable function. The three main types of layers to build ConvNet’s architectures are the convolutional layer, pooling layer, and fully connected Layer. Our layer architecture, as is shown in Figure 5, consisted of:

1. Input layer: converts the input image into mxnxr data usable for the following layers, where m and n are the height and width of the image in pixels, respectively, and r is the depth (e.g., one for gray-scale images and three for Red Green Blue (RGB) images).
2. Convolutional layer: computes the output number of neurons that are connected to local regions of the input image by computing a dot product between their weights and a small region they are connected to in the input. The convolutional layer will have k filters (also called kernels) of size sxtxq where s and t must be smaller than the dimension of the image and q can either be the same as the number of channels r or smaller and may vary for each kernel. In our architecture, we used a 2D convolution composed by k=16 filters and a size of 12 × 12 × 1.
3. ReLU layer: also known as the Rectified Linear Unit layer (ReLU), applies an element-wise activation function, such as max(0,x), to transform the summed weighted input from the node into the activation of the node or output for that input. In other words, it will output the input directly if it is positive; otherwise, it will output zero.
4. Pool layer: This layer will perform a downsampling operation along the spatial dimensions (width and height). For example, in our architecture, the pool size was [2,2], which means that if the layer returns the values $\left[\begin{array}{cc} 1 & 2\\ 3 & 4 \end{array}\right]$, the maximum value in the regions of height two and width two will be selected, which is four.
5. Fully connected layer: The objective of a fully connected layer is to take the results of the convolution/pooling process and use them to classify the image. The fully connected layer goes through its own back propagation process to determine the most accurate weights. Each neuron receives weights that prioritize the most appropriate label.
6. Softmax layer: This layer limits the output of the previous step to classification into the range zero to one. This allows the output to be interpreted directly as a probability.
7. Classification layer: computes the cross-entropy loss for multi-class classification problems with mutually exclusive classes. In other words, it performs the classification based on the output of the previous layer.

To perform the training, we need a solver. Three of the most common are:

1. Stochastic Gradient Descent with Momentum (SGDM) optimizer [24].
2. Root Mean Squared Propagation (RMSProp) optimizer [25].
3. Adaptive moment estimation (Adam) optimizer [26].

## 5. Results

### 5.1. Image Database Creation

Both Machine Learning (ML) algorithms described in Section 4 need a set of images in order to be trained for the later classification. This set of images is called the “training dataset”. The larger the training dataset is, the better the algorithms can be trained. A different set of images called the “testing dataset” was used for testing the classifier and evaluating the classifier’s accuracy. A third type of database, called the “validation dataset”, may be additionally used for validating during the training process, specially using CNN.

In order to create the databases, a large set of independent simulations was carried out, as described in Section 5.2. The entire dataset contained 61,800 binary images (i.e., images were only composed of ones (black) and zeros (white) pixels).

**Figure 6:**
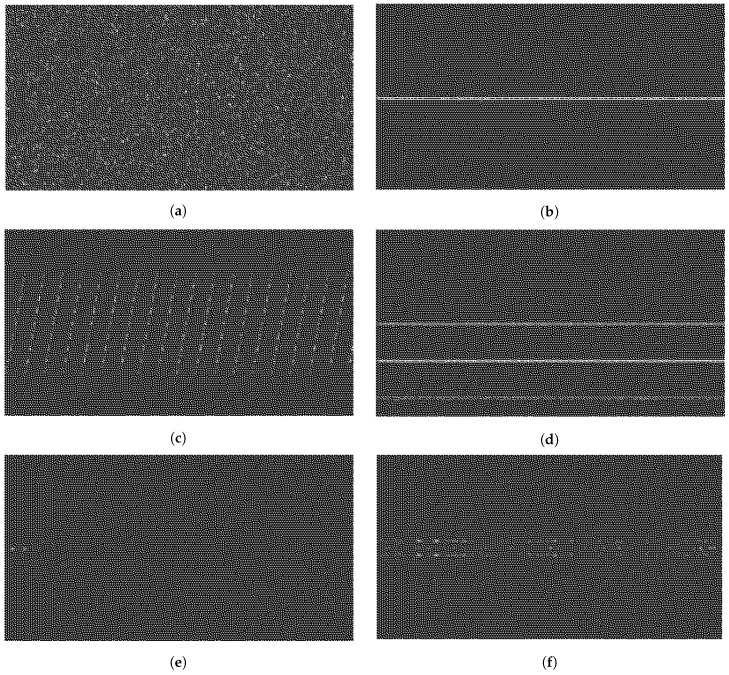
Binary spectrogram images of common jamming signals in the baseband. (**a**) No jammer. (**b**) AM jammer. (**c**) Chirp jammer. (**d**) FM jammer. (**e**) Pulse jammer. (**f**) NB jammer.

The whole database was then split in three parts: 6000 images for training purposes (i.e., 1000 images per jammer type), 1800 images for validation purposes (i.e., 300 images per jammer type), and 54,000 images for testing (i.e., 9000 images per jammer type). Thus, the dataset was split as 10/90%, which means 10% for training/validation and 90% for testing purposes. Figure 6f shows some examples of binary images used in the ML algorithms. The spectrogram looks similar to the ones shown in Figure 1, but in binary scale instead of RGB and with a pixel resolution of 512 × 512. Several resolutions were also tested in our studies, and a 512 × 512 resolution proved to give the best results.

### 5.2. Simulation Parameters

The simulations steps were the following:

- Generating the GNSS signal plus one of the jammer types at a time, using random signal parameters following a certain uniform distribution, as is summarized in Table 1. AM and FM tones were uniformly distributed between 0.1 MHz and 10 MHz. Chirps $T_{\mathrm{swp}}$ and $F_{\mathrm{swp}}$ were uniformly distributed between 5–20 μs and 5–20 MHz, respectively. The bandwidth for NB jammers was set between 20 MHz and 2 GHz. Finally, for the pulse jammer, the duty cycle (τ) and repetition frequency ($F_{r}$) was set between 1–19 μs and 0.1–1.9 THz.
- Sending the GNSS signal (with or without jammer) over a wireless channel with Additive White Gaussian Noise (AWGN). The $C/N_{0}$ and JSR of the GNSS and jammer signals were set as well following a uniform distribution between 25 dBHz and 50 dBHz and 40 dB and 80 dB, respectively (i.e., CN0∼U(25,50) dBHz and JSR∼U(40,80) dB).
- Computing the spectrogram of the received signal and saving it as a black-and-white image in the training, validation, or testing dataset
- Applying the SVM and CNN algorithms described above to classify the test images, based on the information contained in the training and validation image databases.
- Computing the confusion matrix, counted as the percentages of correctly classifying each jammer type (its diagonal values) and the percentages of misclassifying a jammer type (the other values in the matrix except the diagonal values)

The training process with SVM was built on a K-means clustering with a 500 word visual vocabulary and a total number of features of 4,561,920. Not all the features were used to decrease the computational complexity, but only 70% of the strongest features were used. The 70% parameter was again fine-tuned based on experiments.

In terms of kernel choice, we used the RBF kernel since it works well in practice and it is relatively easy to tune. In order to find the optimal hyper-plane, we used the Sequential Minimal Optimization (SMO) method [27], which is an iterative algorithm based on Lagrange multipliers for optimization. It was responsible for solving the Quadratic Programming (QP) during the training of SVM’s.

The CNN was trained during 25 epochs with the Adam algorithm [28] due to its good performance and because it has been specifically designed for training deep neural networks. This means that the entire training dataset was passed both forward and backward through the neural network 25 times using the Adam algorithm in order to find the optimal hyper-plane.

### 5.3. Confusion Matrix Results

**Figure 7:**
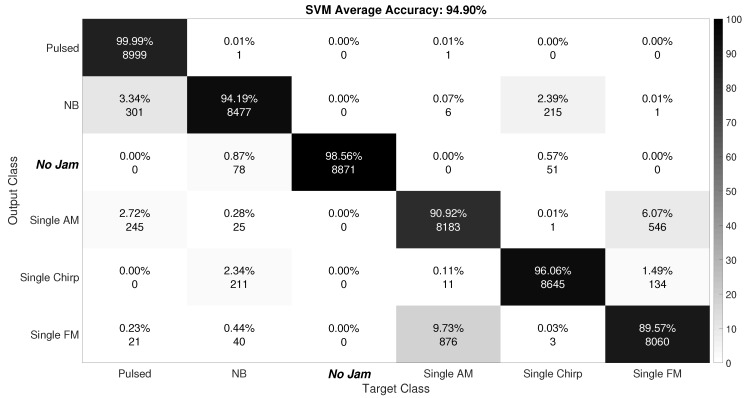
SVM confusion matrix.

**Figure 8:**
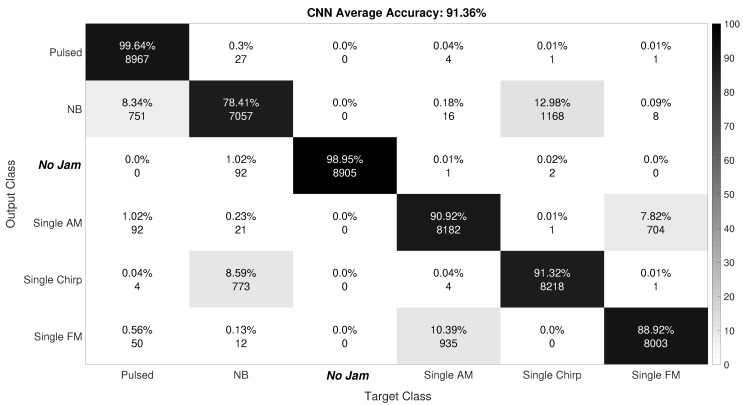
CNN confusion matrix.

Figure 7 and Figure 8 show the confusion matrix for the SVM and CNN algorithms, respectively.

The meaning of the different colors of each label box is summarized in the right side color bar. The confusion matrix shows how accurate a classifier is, in terms of how well it classifies and misclassifies the test dataset in the different classes it has.

Figure 7 shows that the SVM algorithm had a mean accuracy of 94.90%. SVM was able to determine if the jammer was present or not with an accuracy of more than 98%. Only less than 2% of cases were misclassified as the jamming-present scenario when in fact there was no interference. The classification of pulse and chirp jammers offered close to 100% accuracy with the testing dataset used. The accuracy of AM and FM single-tone jammers was around 90%. Finally, we observed that NB jammers were classified correctly almost 94% of the time. These results are really promising, since for example, we can determine the presence of interference with an accuracy of more than 98% or we can determine if it is a pulse jammer with an accuracy of almost 100%. Especially important to notice is that these results were achieved with a training dataset of only 6000 images.

Similarly, Figure 8 shows that the CNN algorithm had a mean accuracy of 91.36%, which was just about 3% smaller than using SVM, but still very high. The reason for the decrease in accuracy in CNN compared to SVM could be the fact that there were more parameters to optimize in CNN than in SVM, and finding rigorous mathematical rules for their optimization is an open challenge. Thus, there might still be the possibility to increase the accuracy of CNN further by an improved optimization of its parameters.

We also noticed that, by using CNN, the accuracy determining the presence of a jammer (versus no jammer case) was slightly higher than for SVM, although only by 0.4%.

The pulse jammers’ classification accuracy was also similar to that of SVM, namely close to 100%. The chirp jammers’ accuracy was more than 91%, but also about 5% lower compared with SVM. The classification of AM and FM jammers showed an accuracy of almost 91% and 89%, respectively (slightly smaller than with SVM). Finally, the poorest classification accuracy was obtained for the NB jammers, where we obtained an accuracy of only 78% (i.e., smaller by about 5% than the SVM case).

## 6. Open-Source Data

The dataset of images used to achieve these results, as well as the MATLAB codes to run the simulations using both ML algorithms are available in open access at Zenodo [28].

## 7. Conclusions

This paper presented a methodology to classify jammer types, treating this as an image classification problem. To do so, a dataset composed of 61,800 different images was generated. The dataset contained different jammer types (including the no-interference scenario), as well as randomly generated parameters for these interference types. The parameters were modeled following uniform distributions in order to contemplate a wide amount of different scenarios. The set of generated images was used to train two different ML algorithms: an SVM and a CNN.

The results showed that with a small library of images and not excessively complex parameters/network layer architectures, we could achieve a pretty high mean classification accuracy of more than 90%; specifically, 94.90% and 91.36%, for SVM and CNN, respectively. In addition, the interference and interference-free scenario accuracy classification was close to 99%, for SVM and CNN, respectively. The highest classification accuracy using both SVM and CNN was when the pulsed jammer was applied. Pulse jammers were correctly classified with an accuracy of more than 99%. This happened because pulse jammers had a spectrogram image more dissimilar with respect to the rest of the scenarios, as can be observed comparing Figure 6e with the rest of the sub-figures in Figure 6. For its part, one of the lowest classification accuracies, even though it was more than 90%, was for AM and FM jammers. On the contrary, as happened with the pulse jammer, AM and FM jammers’ spectrograms looked very alike, as can be observed comparing Figure 6b and Figure 6d. In both cases, the most representative feature was one (or sometimes more than one) horizontal straight line, corresponding to the specific CW frequency tones. For its part, NB jammers were much better classified using SVM than CNN, at least with the specific architecture we used for the CNN.

To summarize, we can say that given the low amount of images used for training the algorithms and given the low complexity used during training, surprisingly good results could be obtained. Further improvements can be focused on increasing the complexity of the training layers so the classification can be more accurate; especially to distinguish AM and FM interferences much better, in addition to including and trying to differentiate between single and multi-tone AM and FM jammers.

## Figures and Tables

**Table 1:** Jammer parameters’ summary.

Jammer Type	Parameter
AM jammer	$f_{J}∼U\left(0.1,10\right)$ MHz
Chirp	$T_{\mathrm{swp}}∼U\left(5,20\right)$μs $F_{\mathrm{swp}}∼U\left(5,20\right)$ MHz
FM jammer	$f_{J}∼U\left(0.1,10\right)$ MHz
NB jammer	Bandwidth∼U(20,2000) MHz
Pulse jammer	τ∼U(1,19)μs $F_{r}∼U\left(1\times 10^{11},19\times 10^{11}\right)$ THz

## List of Acronyms

**AWGN**	Additive White Gaussian Noise
**AM**	Amplitude Modulated
**AWGN**	Additive White Gaussian Noise
**BoF**	Bag of Features
**BoW**	Bag of Words
**CDMA**	Code Division Multiple Access
$C/N_{0}$	Carrier-to-Noise Ratio
**CNN**	Convolutional Neural Network
**ConvNet**	Convolutional Neural Network
**CW**	Continuous Wave
**CWI**	Continuous Wave Interference
**DME**	Distance Measurement Equipment
**DL**	Deep Learning
**FM**	Frequency Modulated
**FFT**	Fast Fourier Transform
**GNSS**	Global Navigation Satellite System
**GPS**	Global Positioning System
**IF**	Intermediate Frequency
**JSR**	Jammer-to-Signal Ratio
**ML**	Machine Learning
**NB**	Narrow Band
**QP**	Quadratic Programming
**RBF**	Radial Basis Function
**RFI**	Radio Frequency Interference
**RGB**	Red Green Blue
**SMO**	Sequential Minimal Optimization
**SVM**	Support Vector Machines
**WB**	Wide Band

## Notes

**Sample Availability:** Samples of the compounds on https://doi.org/10.5281/zenodo.3370934 are available from the authors.
